# Crab in amber reveals an early colonization of nonmarine environments during the Cretaceous

**DOI:** 10.1126/sciadv.abj5689

**Published:** 2021-10-20

**Authors:** Javier Luque, Lida Xing, Derek E. G. Briggs, Elizabeth G. Clark, Alex Duque, Junbo Hui, Huijuan Mai, Ryan C. McKellar

**Affiliations:** 1 Museum of Comparative Zoology and Department of Organismic and Evolutionary Biology, Harvard University, 26 Oxford Street, Cambridge, MA 02138, USA.; 2 Department of Earth and Planetary Sciences, Yale University, New Haven, CT 06520-8109, USA.; 3 Smithsonian Tropical Research Institute, Balboa–Ancón, 0843-03092 Panamá, Panamá.; 4 Department of Biological Sciences, University of Alberta, Edmonton, AB T6G 2E9, Canada.; 5 State Key Laboratory of Biogeology and Environmental Geology, China University of Geosciences, Beijing 100083, China.; 6 School of the Earth Sciences and Resources, China University of Geosciences, Beijing 100083, China.; 7 Biology Department, Duke University, Durham, NC 27708, USA.; 8 Computer Animation and Visual Effects, College of Communication and Design, Lynn University, 2601 North Military Trail, Boca Raton, FL 33431, USA.; 9 Longyin Amber Museum, Xishan District, Kunming 650228, Yunnan, China.; 10 Yunnan Key Laboratory for Palaeobiology, Yunnan University, Kunming 650091, Yunnan, China.; 11 MEC International Laboratory for Palaeobiology and Palaeoenvironment, Yunnan University, Kunming 650091, Yunnan, China.; 12 Royal Saskatchewan Museum, Regina, SK S4P 4W7, Canada.; 13 Biology Department, University of Regina, Regina, SK S4S 0A2, Canada.

## Abstract

Amber fossils provide snapshots of the anatomy, biology, and ecology of extinct organisms that are otherwise inaccessible. The best-known fossils in amber are terrestrial arthropods—principally insects—whereas aquatic organisms are rarely represented. Here, we present the first record of true crabs (Brachyura) in amber—from the Cretaceous of Myanmar [~100 to 99 million years (Ma)]. The new fossil preserves large compound eyes, delicate mouthparts, and even gills. This modern-looking crab is nested within crown Eubrachyura, or “higher” true crabs, which includes the majority of brachyuran species living today. The fossil appears to have been trapped in a brackish or freshwater setting near a coastal to fluvio-estuarine environment, bridging the gap between the predicted molecular divergence of nonmarine crabs (~130 Ma) and their younger fossil record (latest Cretaceous and Paleogene, ~75 to 50 Ma) while providing a reliable calibration point for molecular divergence time estimates for higher crown eubrachyurans.

## Teaser

First Mesozoic crab in amber offers insights into the evolution of true crabs and the colonization of nonmarine environments.

## INTRODUCTION

Transitions from marine to nonmarine habitats are infrequent in most metazoan groups, largely due to different physical and physiological requirements in saltwater and freshwater, competition with previously established residents, and exposure to new predators (*1*). True crabs, or Eubrachyura, are among the animal groups that have conquered land, brackish water, and freshwater multiple times independently, although much more recently than other arthropod groups [e.g., (*2*, *3*)].

Eubrachyurans are the most diverse group of crabs today in terms of anatomy, ecology, and species richness. They fall into two main categories based on the position of their sexual openings: the heterotremes (including primarily and secondarily freshwater crabs), with openings on the legs in males and thorax in females, and thoracotremes (including semiterrestrial and terrestrial crabs), with openings on the thorax in both sexes (*4*–*6*). The consensus is that heterotremes and thoracotremes together form a monophyletic clade Eubrachyura (*7*–*10*). However, two questions, (i) the phylogenetic position of the different freshwater crabs, and (ii) whether thoracotremes are a sister group to heterotremes or derived from within them, are still debated (*7*).

Molecular phylogenies suggest that the first freshwater and terrestrial crabs diverged from their closest marine relatives during the Early Cretaceous or before [~125 million years (Ma) ago] (*8*). Direct evidence of their colonization of nonmarine environments is sparse, however, because the few fossils known from such environments largely correspond to isolated carapaces or claws of latest Cretaceous to Quaternary age (~73 Ma to Holocene) (*11*, *12*). This gap of ~50 Ma between the fossil record of freshwater and terrestrial crabs and their predicted molecular divergence time obscures our understanding of the transition to nonmarine habitats and the number of times such transitions have occurred across families.

**Fig. 1.**
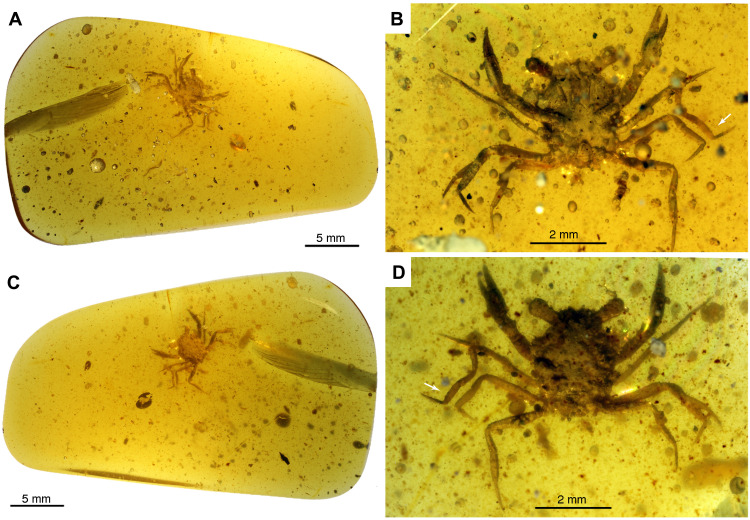
*Cretapsara athanata* Luque gen. et sp. nov., a modern-looking eubrachyuran crab in Burmese amber. (**A** to **D**) Holotype LYAM-9. (A) Whole amber sample with crab inclusion in ventral view. (B) Close-up of ventral carapace. (C) Whole amber sample with crab inclusion in dorsal view. (D) Close-up of dorsal carapace. White arrows in (B) and (D) indicate the detached left fifth leg or pereopod. Photos by L.X. Figure by J.L.

**Fig. 2.**
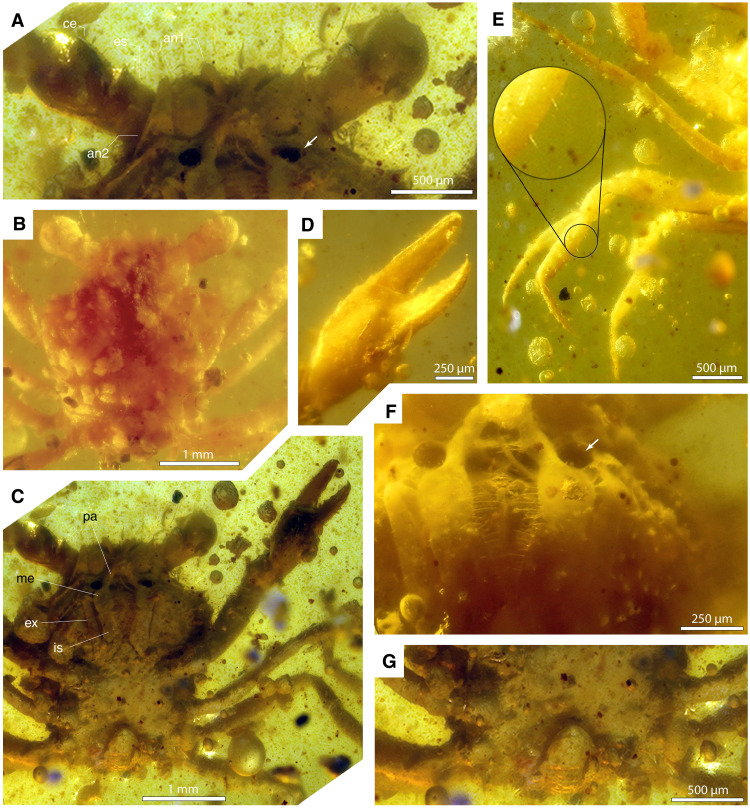
*Cretapsara athanata* Luque gen. et sp. nov. from Burmese amber. (**A** to **G**) Detailed views of Holotype LYAM-9. (A) Close-up of antero-ventral carapace with eyes and eyestalks, antennae, and the excurrent openings beside the palps of the third maxillipeds (mxp3). (B) Close-up of dorsal carapace showing the orbits lacking orbital fissures and the anterolateral margin with three spines including the outer orbital spine. (C) Overview of specimen in ventral view with legs, pleon, sternum, mouthparts, eyes, and left cheliped. (D) Close-up of left cheliped in ventral oblique view, showing a straight dactylus and pollex, the latter bearing small acute teeth on occlusal margin. (E) Ventral view of pereopods 2 to 5, showing all legs of similar size and slender shape, with small, thin, spaced setae, and an acute, long dactyli. (F) Close-up of the mxp3, showing the palp and the fine lining of setae in the occlusal margin, the two hollow openings beside the palp of mxp3 (white arrow), and small granulations in the pterygostome. (G) Close-up of posterior sternites and pleon tucked under the carapace, typical of a eubrachyuran. White arrows in (A) and (F) indicate the excurrent openings. an1, antennula; an2, antenna; ce, corneal eye; es, eyestalk; ex, exopod; is, ischium; me, merus; pa: distal palp of mxp3. Photos by L.X. Figure by J.L.

**Fig. 3.**
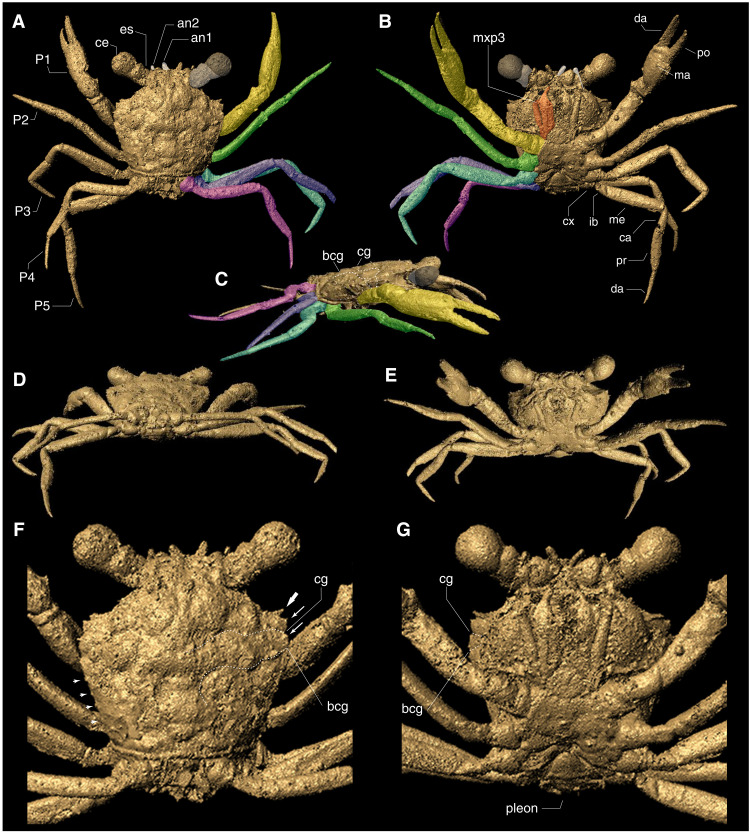
3D mesh of *Cretapsara athanata* Luque gen. et sp. nov. holotype LYAM-9. (**A** to **E**) 3D mesh extracted from reconstructed micro-CT data in VGSTUDIO MAX, remeshed in MeshLab, and visualized using Autodesk Maya: (A) dorsal, (B) ventral, (C) right lateral, (D) oblique postero-dorsal, (E) oblique antero-ventral views, showing the claws of equal size and four pairs of slender legs similar in shape and size, with P5 slightly smaller than the other legs. (**F** and **G**) Details of the dorsal (F) and ventral (G) carapace, showing details of the large eyes and orbits, small antennae, and a small, acute outer orbital spine [(F) thick arrow], two small anterolateral spines (F, thin arrows), a posterolateral margin bearing at least four small and equidistant tubercles (F, small arrows), straight posterior margin, slender coxae of the pereopods, a typical heterotreme eubrachyuran sternum (G), and a reduced and folded pleon with the first pleonites dorsally exposed. Left fifth pereopod digitally reattached. bcg, branchiocardiac groove; ca, carpus; cg, cervical groove; cx, coxa; da, dactylus; ib, ischiobasis; ma, manus or palm of claw; P1, claws or chelipeds; P2 to P5, pereopods or walking legs 2 to 5; po, pollex or fixed finger cheliped propodus; pr, propodus. Images by E.G.C. Figure by J.L.

**Fig. 4.**
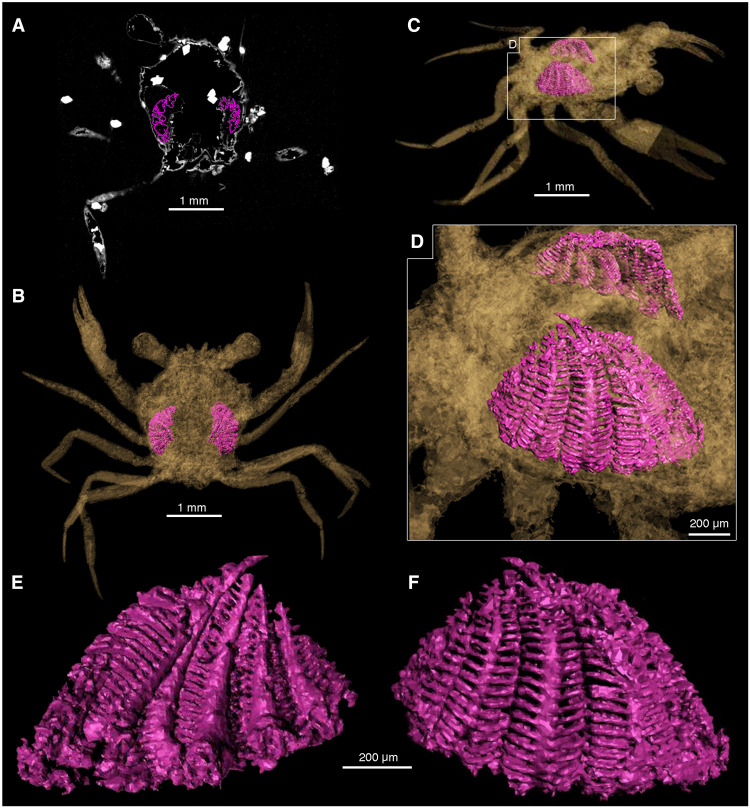
3D reconstructions of *Cretapsara athanata* Luque gen. et sp. nov. holotype LYAM-9 and its gills. (**A**) A single micro-CT slice, coronal section near the base of eyestalks. (**B**) 3D mesh extracted from reconstructed micro-CT data in VGSTUDIO MAX, remeshed in MeshLab, and visualized using Autodesk Maya (see Materials and Methods); dorsal view showing the position of the gills. (**C**) Right lateral oblique view. (**D**) Close-up of (C) showing the right (bottom) and left (top) gills seen from the right side. (**E** and **F**) Close-up of the right gills seen from inside (E) and outside (F). Notice the branchial lamellae and the afferent/efferent vessels. Images by E.G.C. Figure by J.L.

Here, we describe a previously unknown eubrachyuran crab, *Cretapsara athanata*, preserved in Cretaceous amber (~99 Ma, Cenomanian) from Myanmar, Southeast Asia (Figs. 1 and 2). This is the oldest occurrence of a true crab in amber and one of the oldest crown group eubrachyurans known. Micro–computed tomography (CT) digital reconstructions reveal that antennae, large compound eyes, mouthparts with multiple fine hairs, and even gills are preserved (Figs. 3 and 4). Our phylogenetic analysis supports the establishment of a new family with a unique mixture of primitive and advanced characters. The exceptional preservation of *C. athanata* in Cretaceous amber shows that highly carcinized brachyurans (*13*) were already established in nonmarine environments by the earliest Late Cretaceous.

## RESULTS

### Systematic paleontology

Euarthropoda Lankester, 1904

Decapoda Latreille, 1802

Brachyura Latreille, 1802

Eubrachyura Saint Laurent, 1980

Cretapsaridae Luque, fam. nov.

LSID. Family group: http://zoobank.org/urn:lsid:zoobank.org:act:81705335-CAED-400E-B1EC-161B56B298AE.

Included genus: *Cretapsara* Luque, gen. nov., by monotypy.

Diagnosis: As for type genus and species.

**Fig. 5.**
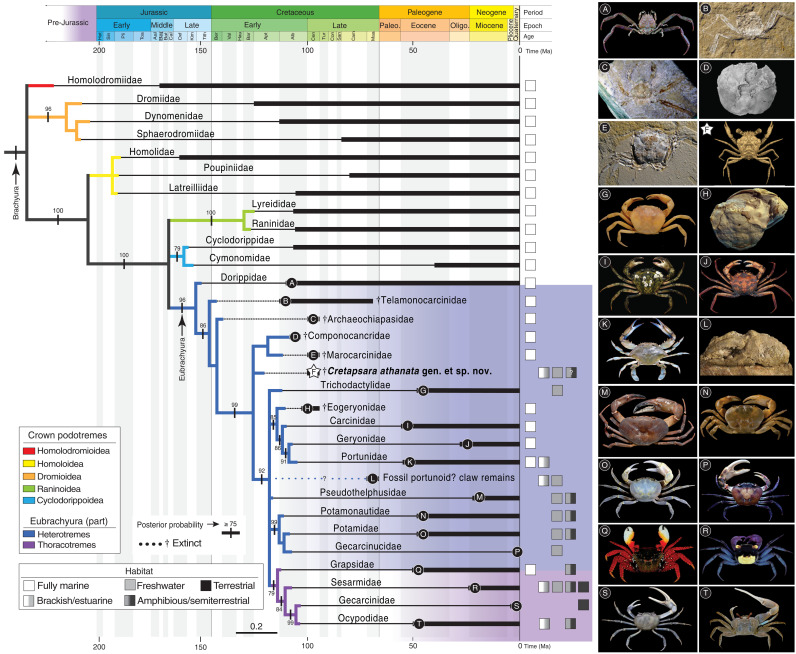
Phylogenetic relationships of *Cretapsara athanata* Luque, gen. et sp. nov. in the crab tree of life. Bayesian majority-rule consensus topology of the post–burn-in sample of trees for key fossil and extant eubrachyuran families, including probability support values indicated at branches. Branches with posterior probability support <75% are collapsed. Thick solid lines represent the ages of the known first and last occurrences of the studied families. Dotted lines and daggers (†) indicate extinct taxa; solid lines indicate living taxa. Photos: *Heikeopsis japonica* (**A**) by T.-Y. Chan; *Telamonocarcinus gambalatus* (**B**), *M. pasinii* (**E**), and *E. elegius* (**H**) by À. Ossó; *Archaeochiapasa mardoqueoi* (**C**) by F. Vega; *Componocancer robertsi* (**D**) by R. Feldmann and C. Schweitzer; *Dilocarcinus septemdentatus* (**G**) and *Melothelphusa dardanelosensis* (**M**) by Senckenberg Museum, S. Tränkner, courtesy of C. Magalhães [(M) after (*80*)]; *Cretapsara athanata* Luque gen. et sp. nov. (**F**) herein; *C. maenas* (**I**), *C. sapidus* (**K**), *Geothelphusa olea* (**O**), *Sayamia melanodactylus* (**P**), *Geosesarma dennerle* (**R**), and *C. guanhumi* (**S**) by O. Radosta; *G. trispinosus* (**J**) by C. Noever (CC BY 4.0, https://commons.wikimedia.org/w/index.php?curid=63236719); *Dinocarcinus velauciensis* (**L**) by N. Robin; *Potamonautes kunduno* (**N**) by P. Crabb (CC BY 3.0, https://commons.wikimedia.org/w/index.php?curid=29254322); *G. cruentata* (**Q**) by A. Anker; and *Leptuca oerdesti* (**T**) by J.L. Figure by J.L.

Remarks: *Cretapsara athanata* n. gen. et sp. differs from any known crab family in its combination of plesiomorphic and apomorphic characters. Superficially, it shares some resemblance to some marine eubrachyuran families such as Eogeryonidae, from the Upper Cretaceous of Spain [*Eogeryon elegius*, Cenomanian (*14*)] (Fig. 5H) and the Lower Cretaceous of Brazil [*Romualdocarcinus salesi*, Albian (*15*)], and Marocarcinidae, from the Upper Cretaceous of Morocco [*Marocarcinus pasinii*, Cenomanian (*16*)] (Fig. 5E). *Cretapsara* differs from Eogeryonidae and Marocarcinidae in the presence of a bilobate rostrum and lack of orbital fissures, compared to the bifid and acute rostrum and two well-developed orbital fissures diagnostic of these two families [Marocarcinidae was initially interpreted as having no orbital fissures due to the erroneous interpretation of the eyestalks as part of the orbit (*16*) but is here recognized as having two orbital fissures and scored as such in our analysis].

The overall carapace outline, the broad front, long legs, and the lack of orbital fissures that characterize *C. athanata* (Figs. 1 and 2) resemble those of some modern Grapsoidea—a group of highly terrestrial thoracotreme crabs common in subtidal and supratidal settings worldwide. Despite this, *C. athanata* differs from grapsoids in important respects. In most grapsoids, there is a conspicuous V-shaped notch between the merus and ischium of the third maxillipeds (mxp3), so that the mandibles are visible through a large, rhomboidal gap when the mxp3 are closed, a feature absent in *Cretapsara* (Figs. 2, C and F, and 3, B, E, and G). Moreover, the podomeres of the walking legs in grapsoids, as in most thoracotreme crabs, are subtriangular to flattened in cross section, whereas those in *Cretapsara* are circular in cross section and more similar to those of other heterotremes (Fig. 3, movie S1, and data file S1). Such similarities between *Cretapsara* and some grapsoids are likely convergent adaptations to similar ecologies.

*Cretapsara athanata* Luque, gen. et sp. nov.

LSID. Genus group: http://zoobank.org/urn:lsid:zoobank.org:act:E6F68190-D2A7-4D6E-8CE8-EDD319E8DA13.

LSID. Species group: http://zoobank.org/urn:lsid:zoobank.org:act:B6E29B76-C37E-47D8-9FDE-228DEB3DB9BA.

Etymology: The genus name combines *Creta* (Latin), for chalk as in Cretaceous, and *Apsara*, a spirit of the clouds and waters; gender is feminine. The specific epithet is based on *athanatos* (Greek), immortal, referring to its lifelike preservation. The family name is based on the only known genus, erected herein.

Holotype: Specimen LYAM-9 (Figs. 1 and 2) deposited in the Longyin Amber Museum, Xishan District, Kunming 650228, Yunnan Province, China. Specimen collected by local miners in 2015.

Type locality and horizon: Angbamo site, Tanai Township, Myitkyina District, Kachin Province, Myanmar [~98.8 ± 0.6 Ma; lowermost Cenomanian (*17*–*19*)].

Diagnosis: The carapace is subhexagonal, nearly as long as it is wide; the orbitofrontal margin is nearly as wide as the body and bears wide, shallow orbits that lack supraorbital fissures or intraorbital spines. The rostrum is bilobate, short, and wide, measuring about half the width of the orbitofrontal margin. The anterolateral margin is one-third as long as the posterolateral margin, and it bears a small outer orbital spine and two short subtriangular anterolateral spines; the posterolateral margin is straight to slightly convex, and it bears at least four small and equidistant tubercles. The posterior margin is wide, nearly straight, rimmed, and apparently ornamented with a row of small tubercles. The cervical and branchiocardiac grooves are well developed, reach the lateral margins of the body, and are expressed ventrally; the dorsal regions are well developed and delimited by grooves. The buccal cavern is wide, covered by a pair of operculiform mxp3 that lack a V-shaped incision in the occlusal margin. The thoracic sternites 1 to 3 are visible ventrally and are separated from a subtrapezoidal sternite 4 by a shallow groove. Sternites 5 and 6 are similar in shape; sternite 5 is the widest of all, and sternites 6 to 8 reduce in size posteriorly. The sterno-abdominal cavity is shallow, and there is no evidence of a linea media traversing the sternites. The pleon has a small subtriangular telson and, based on the stereomicroscope images and the micro-CT scanning, apparently has six free and unfused pleonites, from which at least the first two are fully visible dorsally; there is no evidence of uropods or uropod remains. The eyes are as large as the orbits; the corneal eye is globular, as wide as it is long, and apparently covered by small hexagonal facets; the eyestalk is short and cylindrical. The antennulae and antennae are reduced. The claws or chelipeds are equal in shape and size, and the four pairs of walking legs are well developed, slender, and similar in size and shape; they lack chelate, subchelate, or flattened distal podomeres (e.g., propodi and dactyli); the podomeres are semicircular in cross section. Excurrent openings are well developed, small, and circular.

Description: *Dorsal carapace:* The carapace is subhexagonal in outline and about as long as wide (~2 mm) (Figs. 1D and 2B). The orbitofrontal margin is nearly as wide as the maximum width of the carapace, and each orbit is wide, concave, and shallow, with a sinuous supraorbital margin that lacks supraorbital fissures or spines (Figs. 2, A to C, and 3), apart from a small, anterolaterally diverging outer orbital spine (Fig. 3, F and G, thick arrow). The rostrum is short and half the width of the orbitofrontal margin, downturned, apparently with a shallow axial sulcus and raised subparallel lateral margins grading into the inner orbital margins (Figs. 2B and 3, A and F, and movie S1). The anterolateral and posterolateral carapace margins are distinct and separated by a shallow lateral expression of the cervical groove (Fig. 3, F and G, dotted line). The anterolateral margin is slightly convex, about one-third the length of the posterolateral margin, and it bears two anterolateral spines that are short and subtriangular (Figs. 2 and 3F). The edge of the posterolateral margin is straight to slightly convex, and it bears at least four small tubercles that are somewhat equidistant from each other (Fig. 3F, movie S1, and data file S1). The posterior margin of the carapace is about two-thirds of the maximum carapace width, lacks reentrants, and, as evidenced by the micro-CT scans, appears to be rimmed and bearing a row of small tubercles (movie S1 and data file S1).

The cervical groove becomes faint axially, deepens laterally reaching the anterolateral margin, and is expressed ventrally (Fig. 3, C, F, and G); the groove is concave posterior to the mesobranchial region and sinuous toward the lateral margin. The branchiocardiac groove is well developed, somewhat deep, flanking the cardiac region and, apparently, the urogastric region mesially and the mesometabranchial region distally (Fig. 3F).

The epigastric region is wide, lacking spines or tubercles, laterally inflated, and axially sulcate; the lateral lobes reach the frontal region and are nearly continuous posteriorly with the protogastric lobes (Fig. 3, A and F). The protogastric region is wide, lacking spines or tubercles, laterally tumid, and forming an almost ovate longitudinal lobe flanked by the epigastric region; axially, the protogastric depression separates the protogastric lobes and opens to the narrow anterior mesogastric region. The mesogastric region is relatively small, lacking spines or tubercles, defined anteriorly by two grooves that separate the protogastric lobes and posteriorly by a concave cervical groove. The metagastric region is well developed, inflated, and about three times as wide as long; it is bounded anteriorly by the cervical groove, laterally by grooves that separate it from the epibranchial regions, and posteriorly by a narrow and depressed urogastric region. The urogastric region is considerably shorter and narrower than the metagastric region, about twice as wide as it is long, depressed relative to the metagastric and cardiac regions, and separated from them by faint grooves, delimited laterally by the branchiocardiac groove. The cardiac region is almost subtrapezoidal, lacking spines or tubercles, is wider posteriorly, and is delimited laterally by the branchiocardiac groove. The intestinal region appears to be wide, shorter mesially, and slightly wider laterally, with two lateral swellings parallel to the posterior margin (Fig. 3, A and F, and movie S1).

The postorbital region is tumid, is separated from the protogastric lobes by a deep and wide groove, and is bounded posteriorly by the cervical groove and laterally by the hepatic region; the postorbital region lacks spines or tubercles. The hepatic region is small, is flat to slightly depressed, and is bounded laterally by the anterolateral margin and posteriorly by the cervical groove. The epibranchial region is wide and is delimited anteriorly by the cervical groove and posteriorly by a shallow groove that separates the epibranchial and mesometabranchial regions; the epibranchial region has a swollen lobe between the anterolateral margin and the metagastric region. The mesobranchial and metabranchial regions are undifferentiated, smooth, and unornamented (Fig. 3, A and F, and movie S1).

*Ventral carapace:* The buccal cavern is wide and covered by a pair of operculiform mxp3. The coxae of the mxp3 separate the sternum from the pterygostome. The pterygostome is curved, bulged, finely granulated, with a row of coarser tubercles parallel to the linea brachyura or molting plane (Figs. 2, C and F, and 3, and movie S1).

The thoracic sternum has fused sternites 1 to 3 that are triangular, about as long as wide, and are visible ventrally; sternite 4 appears to be subtrapezoidal in outline; sternite 5 is the widest and measures approximately two-thirds of the maximum carapace width; sternite 6 is narrower than sternite 5 but nearly as wide as sternite 4; sternites 7 and 8 are small and barely evident in the micro-CT scan. The sterno-abdominal cavity is shallow, and there is no evidence of a linea media traversing the sternites.

The pleon, based on the micro-CT scan reconstruction, appears to comprise six free, unfused pleonites and a small telson. The pleonites are subrectangular in outline. The first pleonite is smallest and visible dorsally, and the second is wider and, apparently, also visible dorsally. The third and fourth pleonites are the widest, with the anterior part of the third pleonite visible dorsally, but the fourth is concealed under the body. The fifth pleonite is slightly narrower than the fourth sternite, whereas the sixth pleonite is considerably narrower than the preceding pleonites. The telson is triangular and about as wide as long. There is no visible evidence of uropods.

*Eyes and antennae:* The eyes and eyestalk are large, with each eye approximately one-third the maximum carapace width. The corneal eye is globular, as wide as long, measuring about one-fifth the width of the carapace, and seems to be covered with small hexagonal facets; the eyestalk is shorter and cylindrical (Figs. 2, A to C, and 3, B, E, and G). The antennula and antenna are very small (Figs. 2, A to C, and 3) and, due to their small size and limitations in the resolution of the micro-CT scans, no details of the flagella can be discerned.

*Mouthparts and thoracic appendages:* The mxp3 consist of a long slender exopod and an endopod with a long semirectangular ischium, a squarish merus, and slender distal palp (Figs. 2, A, C, and F, and 3, B, E, and G). The coxae of the mxp3 are small and do not meet axially; the ischiomerus has straight occlusal margins lined with fine setae (Fig. 2, A, C, and F) and does not form a rhomboid gap with the other mxp3 that reveals the other mouthparts, neither does it bear a crista dentata (Figs. 2, C and F, and 3, B, E, and G). The palp, which is formed by the propodus, carpus, and merus, is long, thin, and located in an inner-mesial position.

The first of the five pairs of pereopods (P1) constitutes the claws (or chelipeds), which are symmetrical or homochelous (Figs. 1 and 3). Their mobile fingers (or dactyli) are long and slender, barely curving downward, and parallel to the fixed finger or pollex of the propodus, which is also long and slender and apparently bears six to eight very small teeth on the occlusal margin (Fig. 2D, movie S1, and data file S1). The other four pairs of thoracic appendages are slender walking legs (P2 to P5), similar in size and shape, with slender, acute dactyli and lacking chelate or subchelate terminations (Figs. 1 and 3). The podomeres are semicircular in transverse section rather than flattened (Figs. 1 to 4, movie S1, and data file S1). The claws and the legs have several small, fine setae scattered over their surface (Fig. 2D and E).

*Gills and excurrent openings:* The micro-CT scan of the holotype of *C. athanata* revealed the presence of at least six pairs of phyllobranchiate gills, bearing apparently up to 20 series of well-developed, flattened branchial lamellae that are perpendicular to the main gill shaft (Fig. 4, D to F, and movies S2 to S4). A pair of conspicuous, small, and subcircular excurrent openings is evident beneath the antennal sockets adjacent to the epistomial region (Figs. 2, A, C, and F, and 3, B and G; movie S1; and data file S1).

## DISCUSSION

### Taphonomy

*C. athanata* is the first crab discovered in Mesozoic amber. The holotype is complete and articulated and preserves delicate features such as antennae, compound eyes, and mouthparts covered in minute hairs or setae (Fig. 2), which are rarely preserved in fossil crabs. The overall posture of the legs and claws (Figs. 1 to 4), the fully articulated posterior carapace and pleon (Fig. 3, A, C, D, and F), the delicate mouthparts barely open (Figs. 1 to 3), and the intact molting line (linea brachyura) and pterygostome in place (Figs. 2C and 3, B, C, E, and G; and movie S1) indicate that the holotype was entombed in resin while still alive. Although there are no struggle marks evident within the surrounding amber (e.g., drag bubbles), the pereopod 5 on the left side of the body has detached at the base of the coxa (Fig. 1, white arrows), possibly indicating limb autotomy as the crab was engulfed before the resin polymerized.

Milky amber is present along the ventral surface of the carapace adjacent to the sternites and, to a lesser extent, around the maxillipeds (Fig. 2, F and G). It is unclear whether this might be the result of decay products or moisture from the gill cavity interacting with the surrounding resin to form microscopic bubbles within the amber (*20*). The presence of small euhedral pyrite crystals inside the limbs and carapace (Fig. 4A, white masses) indicates the availability of iron and the activity of sulfate-reducing bacteria within the decaying carcass or surrounding sediment. Sulfate is present in elevated concentrations within seawater, and pyrite may indicate proximity to coastal or brackish settings (*21*). However, sulfate may also be sourced by weathering or exposure to marine water brought inland due to tropical storms or flooding events. The growth of these crystals in voids within the exoskeleton and also in vacuoles within the surrounding amber suggests that pyrite formation occurred late in the taphonomic process and may provide more information about diagenetic conditions and porewater chemistry than about the biostratinomy or habitat of the crab (*22*).

Although there are no other arthropod syninclusions associated with the crab to guide interpretation of the preservational setting and nearby habitats, the amber appears to be a single flow of resin that contains numerous particulate inclusions and bubbles. Among the particulates are numerous carbonized wood fibers (Fig. 2): Some of these clumps of dark brown or black plant tissue show cell structure, whereas others appear to represent masses of insect frass. Such features are associated with litter amber that is formed on or near the forest floor (*23*). Similar organic particulates have been found alongside inclusions from marine habitats in Burmese amber, such as an ammonite shell and marine snails (*24*) and a marine ostracod (*25*). They have also been found alongside microfossils that were blown into Cretaceous resin from coastal habitats (*26*, *27*) and shrimps that were trapped in a predominantly freshwater setting (*28*). The amber containing the crab differs from previously recovered aquatic samples, however, in that it does not contain beach sand [e.g., (*24*)], it lacks distinct layers within the amber that appear to alternate between terrestrial and aquatic resin flow conditions [e.g., (*28*)], and it contains a carcass instead of an empty shell or molt (e.g., (*27*)]. Thus, it is likely that *C. athanata* was entombed in a freshwater or brackish setting near the amber-producing forest—not in a fully marine setting where resin is exposed to aquatic organisms through variations in water levels or winds. On the basis of the current sample size and information available, the possibility that *C. athanata* was fully terrestrial cannot be ruled out, although an amphibious lifestyle is more likely given that several extant species of primarily freshwater crabs are amphibious.

### Ontogeny and life stage

The holotype of *C. athanata* (carapace 2 mm wide, leg span 5 mm) is considerably smaller than most other known crab species—fossil or extant, marine or nonmarine (Figs. 1 to 4). Body sizes of just a few millimeters long are typical of early ontogenetic stages in crabs [i.e., megalopae larvae and early postlarval juveniles (*29*)] but tend to become much larger in subsequent molts. However, some species with miniature adults are known among extant spider crabs and pea crabs (mostly marine) (*30*, *31*), while extinct, pedomorphic (*7*), and “dwarfed” (*32*–*34*) species are known from the early Late Cretaceous and the Cretaceous-Paleogene interval, respectively. A number of diagnostic characters indicate that the specimens of *Cretapsara* described here are postlarval stages: (i) the pleon is folded beneath the cephalothorax; (ii) the sterno-abdominal cavity is shallow; (iii) setal tufts at the tip of the dactyl of the last leg pair (P5), which are typical of crab megalopae, are absent; and (iv) there is no evidence of uropods. However, it is not clear whether the holotype of *Cretapsara* represents a minute adult or the juvenile of a species for which adults have yet to be discovered.

### Gill anatomy

Micro-CT scan images of the holotype of *C. athanata* revealed the preservation of complete gill lamellae (Fig. 3 and movies S2 to S4). To date, fossilized gills in crabs have only been reported in *Romaleon franciscae*, a marine cancroid from the Miocene of the Kerguelen Islands (*35*). Those specimens, preserved in concretions a few centimeters long, have phosphatized gill tissues interpreted as the result of early diagenesis acting on the crab carcasses (*35*). Although the delicate cuticle of the gills can be present in crab exuviae before degradation, the completeness, preservation, and posture of *C. athanata* indicate that the specimen is a carcass and not a molt (see the “Taphonomy” section).

Gills play a crucial role in a number of physiological processes including osmoregulation, ion mobilization, excretion, and respiration [(*36*) and references therein]. As such, the shape, size, and number of gills may vary across crabs with different lifestyles. Unfortunately, the nature of the gills among most groups of living crabs is incompletely known (*4*), and comparisons across a wide range of taxa and habitats are unavailable. Brachyuran crabs have phyllobranchiate gills that are characterized by a flattened gill shaft with one dorsally flowing afferent branchial sinus and one ventrally flowing efferent sinus; these two vessels are connected anteriorly and posteriorly by the gill lamellae (*4*). The six pairs of gills clearly recognizable in the holotype of *C. athanata* are phyllobranchiate, bearing apparently up to 20 well-developed, flattened branchial lamellae perpendicular to the main gill shaft (Fig. 4, D to F, and movie S4).

In fully marine and freshwater crabs, the gills tend to be well developed and occupy most of the branchial chamber, whereas the gills of terrestrial and semiterrestrial crabs may be reduced in size and number, leaving room for the secondary development of a vascularized “lung”-like spongy tissue to facilitate respiration (*4*). As a consequence, terrestrial and semiterrestrial crabs may have wider, sometimes inflated, branchial chambers to accommodate this spongy tissue (*37*). The gills of *Cretapsara*, however, are not reduced, and the branchial regions are too narrow to house complementary lung-like structures (Fig. 3, movies S2 to S4, and data file S1). Furthermore, the excurrent openings in *Cretapsara* (Fig. 2, A, C, and F) are unlike those of modern land crabs. They are small subcircular openings beneath the antennal sockets adjacent to the epistomial region, similar to those in freshwater crabs. Thus, there is no evidence that *Cretapsara* was fully land-dwelling, but most likely water-dwelling or facultatively amphibious, as are several extant freshwater crabs (Fig. 5).

### Amber fossil assemblage

Land-dwelling insects are the most common syninclusions in Cretaceous Burmese amber, but rarer terrestrial arthropods such as oniscidean isopods *Myanmaroscus deboiseae* (*38*) and *Palaeoarmadillo microsoma* (*39*), acari, spiders, and millipedes [e.g., (*40*, *41*) and references therein] also occur, many of them associated with humid forest floor habitats and “litter amber” (*23*). Aquatic invertebrates have also been reported in Burmese amber, but predictably, these are much rarer. They include marine myocopid and brackish-to-freshwater podocopid ostracods [e.g., (*25*, *42*)], marine and land snails (*43*, *44*), and even fragmented and transported empty ammonite shells (*24*). Fragments of crinoid column ossicles, corals, and oysters have been found attached to the outer surface of pieces of amber (*45*, *46*), and borings made by pholadid bivalves are common within this amber deposit (*21*). Adding to the list of aquatic fauna trapped in Burmese amber, freshwater penaeoid shrimp associated with freshwater penny larvae (Psephenidae) have been reported from the area (*28*). These occurrences suggest that the resin-producing forest where *C. athanata* lived, presumably rich in Araucariaceae (*47*) or Cupressaceae (*48*), was close to fluvio-estuarine (*28*) and coastal (*24*) settings.

### Paleoecology

All previous crabs reported from amber are representatives of the family Sesarmidae from the early Miocene of Chiapas, Mexico (~15 Ma) (*49*–*52*). Extant sesarmids are land- and tree-dwelling thoracotreme crabs from tropical to subtropical coastal systems worldwide, some of which live in phytotelms or small water bodies in rainforests (Fig. 5R) (*53*, *54*). Such a lifestyle facilitates the inclusion of sesarmid crabs in tree resins (*49*–*51*).

The available evidence suggests that *C. athanata* was neither marine nor fully terrestrial but a fully brackish to freshwater crab, even facultatively amphibious (movie S5), that lived either on the forest floor or in shallow bodies of water on the forest floor. Most living eubrachyuran families are fully marine (Fig. 5, white squares), but just over a dozen have representatives that have invaded freshwater or moved onto land—several of them independently (*55*).

Extant members of primarily or true freshwater crabs are restricted to the superfamilies Trichodactyloidea, Pseudothelphusoidea, Potamoidea, Potamonautoidea, and Gecarcinucoidea (Fig. 5, light gray squares). They are exclusively freshwater, and their larvae undergo direct development. Some of these species are facultatively terrestrial or even arboreal, reaching small water bodies formed in rainforest trees and epiphytes (*54*, *56*), as do some sesarmid crabs. Secondarily freshwater crabs, such as some varunids, live in freshwater as adults but, unlike primarily freshwater crabs, their free-living larval stages rely on the ocean (*55*). Land-dwelling eubrachyurans, either intertidal or fully terrestrial, are largely restricted to thoracotreme families within Grapsoidea and Ocypodoidea (Fig. 5, Q to T, dark gray and black squares). Obligate air-breathing land crabs such as the Christmas Island red crab, *Gecarcoidea natalis*, undergo massive migrations to the coast to release their planktonic zoeae larvae in the ocean. After 3 to 4 weeks of development, they return to the shoreline by the thousands as new megalopae recruits, which metamorphose into early postlarval juveniles before migrating inland where the adults dwell (*57*). Similar patterns of megalopae and juvenile postlarvae recruitment occur in other true land crabs, such as the gecarcinids *Gecarcinus* and *Johngarthia* [e.g., (*58*)], and in semiterrestrial crabs, such as the sesarmid *Chiromantes* spp. [e.g., (*59*)]. Although the available evidence suggests that *C. athanata* was neither fully terrestrial nor semiterrestrial, unlike the groups mentioned above, it could provide an analog to modern primarily freshwater crabs or even have been a catadromous freshwater crab with marine larval development, whose early postlarvae were trapped in fluvio-estuarine to coastal settings.

### Phylogenetic and evolutionary implications

#### The origins of Eubrachyura

The discovery of *Cretapsara* in amber of early Cenomanian age (~99 Ma), together with the occurrence of several other Cenomanian heterotreme eubrachyurans such as *Eogeryon* from Spain (*14*), *Lithophylax* from France (*60*), and *Marocarcinus* from Morocco (Fig. 5) (*16*), shows that crown eubrachyurans were already diverse worldwide and occupied novel habitats, including freshwater, by this time. Molecular phylogenies indicate that heterotremes and thoracotremes diverged in the latest Jurassic–earliest Cretaceous (175 to 100 Ma) (*8*, *9*). However, the oldest unequivocal heterotremes, which are all marine, are from the latest Early Cretaceous (Aptian-Albian, ~115 to 110 Ma) [e.g., (*7*, *15*, *61*–*63*)]. In contrast, the oldest unequivocal thoracotremes, which are also marine, are from the Eocene (~50 Ma) (Fig. 5) (*52*, *64*, *65*). Unfortunately, most of these early occurrences are so incomplete that their systematic affinities remain unclear, and they are therefore unreliable for node calibrations and inferences about the origins of modern eubrachyurans. Despite the superficial resemblance of *C. athanata* to some thoracotremes (e.g., sesarmids), the form of its cylindrical limb podomeres, the narrow sternum, the visibility of the first pleonites dorsally, and the marked grooves in the dorsal carapace are similar to those in several crown eubrachyuran body plans. Our phylogenetic results support the heterotreme affinities of *Cretapsara*, which is recovered well nested within the heterotremes and closer to the clade formed by most crown eubrachyurans (Fig. 5).

Recent phylogenomic studies indicate that Potamidae, a freshwater family of heterotreme crabs, are sister to Thoracotremata—the group that includes the true land crabs and allies—rather than being closer to other freshwater heterotremes (see also Fig. 5) (*10*, *66*, *67*). Our results support the paraphyly of “Heterotremata,” with thoracotremes nested within heterotremes (Fig. 5, purple clade). This placement supports previous hypotheses [e.g., (*6*)], in which Heterotremata is a synonym of Eubrachyura, and includes thoracotremes as a highly derived heterotreme group. On the other hand, if extinct and extant heterotremes form a monophyletic Heterotremata sister to a monophyletic “Thoracotremata,” then the most recent common ancestor of both clades must have been either a heterotreme or a thoracotreme. This raises the question of the relationships between total-group Eubrachyura and their closest noneubrachyuran relatives, e.g., the crown podotremes Raninoida and Cyclodorippoida that, together with Eubrachyura, form a clade well supported by morphological, molecular, and phylogenomic studies (Fig. 5) [e.g., (*7*, *8*, *66*–*68*)]. As such, the earliest eubrachyurans may have diverged from a common podotreme-like ancestor shared with raninoids and cyclodorippoids but not with other podotremes such as homoloids, dromioids, and homolodromioids.

#### The colonization of nonmarine habitats by true crabs

Exceptionally preserved fossils such as *C. athanata* from early Cenomanian (99 Ma) Burmese amber are crucial to better constrain the timing of incursions into nonmarine habitats by true crabs during the Cretaceous (*7*, *69*), as well as the origination of modern eubrachyurans and fully carcinized forms, i.e., crab-like body forms with a wide and flat carapace, well-developed legs, a reduced muscular pleon folded under the body, and reduced to absent uropods (*13*). Molecular phylogenetic studies suggest that crown Thoracotremata—the group containing semiterrestrial and terrestrial crabs—diverged from other eubrachyurans during the Early Cretaceous (*8*). Unfortunately, the oldest known putative thoracotremes are all marine and Cenozoic in age and consist of carapaces from the lower Eocene (~50 Ma) of Italy, assignable to several families within Grapsoidea (*64*, *65*). In contrast, the oldest confirmed records of terrestrial, semiterrestrial, and arboreal thoracotreme crabs are all Miocene (~22 to 10 Ma) [e.g., (*50*–*52*, *70*)].

Molecular phylogenetics have estimated that primarily freshwater heterotreme crab groups diverged from other heterotremes in the Early Cretaceous (~130 Ma) (*8*), but reconciling these ages with the fossil record is problematic due to its sparse and fragmentary nature. The oldest fossils of crown freshwater crabs come from the Eocene (~50 Ma) of Italy [Potamidae, a dorsal carapace (*11*)] and Peru [Trichodactylidae, isolated claw remains (*12*)] and from the Oligocene (~25 Ma) of Tanzania [Potamonautidae, a dorsal carapace and chelipeds (*71*)] and Brazil [Trichodactylidae, isolated claw remains (*12*)]. The occurrence of *Cretapsara* ~100 Ma in Burmese amber bridges the gap between the estimated molecular divergence time of nonmarine crabs and their Cretaceous fossil record. Molecular studies show that crown groups of primarily freshwater crabs may have evolved at least three times independently: (i) in the African and Eurasian heterotreme clade formed by Gecarcinidae + (Potamidae + Potamonautidae) (Fig. 5, N to P) and likely sister to Thoracotremata (*8*), (ii) in the Neotropical family Pseudothelphusidae (Fig. 5M), and (iii) in the Neotropical family Trichodactylidae, which might be closer to portunoids than to other Neotropical freshwater crabs (*8*). Thus, *C. athanata* may represent a fourth independent incursion of brachyuran crabs into nonmarine habitats.

Isolated claws of fluvio-lacustrine crabs showing possible affinities with xanthoids or portunoids occur in association with dinosaur bones in the Upper Cretaceous (late Campanian, 74 to 72 Ma) of France (*72*) and the United States (Milner and Luque, personal observation) (Fig. 5L). Unfortunately, the taxonomic affinities of these specimens are unclear as claw morphology tends to converge with similar dietary habits across groups, and associated carapaces are unknown. These claw remains cannot be assigned with confidence to any crown freshwater group, and thus, they may represent at least one additional incursion into freshwater habitats by crabs during the Mesozoic. Other secondary nonmarine incursions include freshwater and cave-dwelling crabs within otherwise mainly marine families such as Chasmocarcinidae, Hymenosomatidae, Hexapodidae, and Portunidae [e.g., (*55*, *73*–*75*)]. Brackish waters, estuarine systems, and the intertidal zone, which are transitional between open marine and fully land and freshwater habitats, must have played a crucial role in the evolution of terrestrial, semiterrestrial, and freshwater crabs during the Cretaceous Crab Revolution and the onset of modern clades and body plans (*7*, *69*).

## MATERIALS AND METHODS

### Experimental design

The main objective of this study was to fully characterize LYAM-9 and place it in a phylogenetic context as a basis for interpreting the habitat and preservational history of the specimen. To fully describe LYAM-9 and provide characters for phylogenetic analysis, we used approaches ranging from standard macro- and microscopic observations of external structures, to x-ray micro-CT scanning and observations of 3D models.

### Materials and imaging

The amber sample studied (Longyin Amber Museum, Xishan District, Kunming, Yunnan Province, China) measures 31.5 mm by 19.8 mm by 7.0 mm, weighs 3.05 g, and contains one complete, small brachyuran crab specimen, holotype LYAM-9. The specimen was tested by the National Gemological Training Center in China, and all of its features are consistent with Burmese amber from the Hukawng Valley in the Kachin State of Myanmar. This published work and the nomenclatural acts have been registered in ZooBank, the official registry of Zoological Nomenclature. The LSID for this publication is http://zoobank.org/urn:lsid:zoobank.org:pub:F2CC5291-0DE0-4B21-AB16-9F7B570BAD8E.

The age of the Burmese amber coming from the region where holotype LYAM-9 originates has been estimated as late Albian–early Cenomanian (latest Early Cretaceous to earliest Late Cretaceous, ~105 to 95 Ma), on the basis of biostratigraphic evidence (ammonites and pollen) from the Angbamo site (*17*, *18*). This estimate is consistent with radioisotopic data (U-Pb) from volcaniclastic zircons attached to amber clasts, which indicate a maximum age of 98.8 ± 0.6 Ma (*19*) for the matrix surrounding amber in the region, but not necessarily the amber it contains, which could be either of the same age as the matrix that contains it or slightly older if they have been eroded and redeposited in younger strata.

Specimen microphotography and observations were conducted with a Leica SZ 12.5 stereomicroscope equipped with a Canon EOS Rebel T6i camera and a Z-stacking imaging system, which consists of a computer-driven stage with a Canon EOS 5D DSLR camera equipped with a Canon MP-E 65-mm lens and studio lighting. Submersion in glycerin was used to reduce refraction and optical distortions due to the curvature of amber surfaces.

The amber inclusion was scanned with a Micro–X-ray–CT Xradia 520 Versa (Carl Zeiss X-ray Microscopy Inc., Pleasanton, USA) at the Yunnan Key Laboratory for Palaeobiology, Yunnan University, Kunming, China. The entire amber piece was scanned with a beam power of 50 kV/4 W, with the LE3 filter for 390 min. The exposure time of each projection was 5.5 s. The voxel size was 8.35 μm. The sample was rotated 360° during the scan, and a total of 3201 projections were acquired. All of the projections were used for reconstruction through the automatic reconstruction function of the instrument’s own software (Zeiss Scout-and-Scan Control System 11.1.7859). The center shift (pixels) was 2.216, bean hardening correction constant was 0.05, and smooth constant was 0.5. From the reconstruction, a total of 1012 radiographs were registered and saved as TIFF images for further processing. On the basis of the obtained image stacks, structures of the specimen were rendered using Drishti 2.6.4 and Amira 5.4 (Visage Imaging, San Diego, USA). The subsequent volume rendering and animations were performed using VGSTUDIO MAX 3.0 (Volume Graphics, Heidelberg, Germany). The surface morphology of the specimen was extracted as a watertight polygonal mesh and imported into MeshLab where small isolated polygonal faces were removed. We digitally removed the objects (bubbles and particulates) obscuring the dorsal view of the specimen using MeshLab. Subsurface polygons of the mesh of the left eye and the mesh of the tip of the left claw were removed, and the remaining faces were remeshed via the Screened Poisson surface reconstruction technique to fill holes and smooth the surface. The original and the new meshes of the eye and the claw were imported into Maya (Autodesk, San Rafael, USA), where they were superimposed. Copies of the eye, claw, and right pereopod 5 mesh were reflected across the body plane in Maya and repositioned to create the right eye, the right claw, and the detached left leg. The surface morphology of the gills was extracted in the same manner as the rest of the body; small isolated polygonal faces were removed using MeshLab.

### Phylogenetic analysis

The dataset, containing 30 taxa and 93 adult morphological characters, was built in MorphoBank (data available at http://morphobank.org/permalink/?P4044), modified from (*7*). Undetermined characters and those not preserved were scored as “?,” and inapplicable characters were scored as “–.” Multiple character states present in a given terminal were scored as polymorphisms.

Bayesian inference was used on the dataset, as implemented in MrBayes v. 3.2.6 (*76*). The dataset was analyzed under the traditional Mk model (*77*) with an ascertainment bias correction to account for scoring only variable morphological characters. Each analysis was performed with two independent runs of 3 × 10^6^ generations each. We used the default settings of four chains (one cold and three heated) per independent run. The relative burn-in fraction was set to 50%, and the chains were sampled every 1000 generations. We set the temperature parameter to 0.01 as determined by preliminary runs to achieve chain mixing values in the optimal range (0.4 to 0.8). Convergence of independent runs was assessed through the average standard deviation of split frequencies (ASDSF << 0.01) and potential scale reduction factors [PSRF ≈ 1 for all parameters (*78*)]. We used Tracer v.1.6 (*79*) to determine whether the runs reached stationary phase and to ensure that the effective sample size for each parameter was greater than 200. Results of the Bayesian runs were summarized as a majority-rule consensus tree of the post–burn-in sample with a node support threshold of 75% (nodes with posterior probability support <75% were collapsed).

## Notes

View/request a protocol for this paper from *Bio-protocol*.
