# Supplementary material for: Crab in amber reveals an early colonization of nonmarine environments during the Cretaceous
Source: Sci Adv. 2021 Oct 20;7(43):eabj5689. doi: 10.1126/sciadv.abj5689 (PMC8528423; doi:10.1126/sciadv.abj5689)
Supplement: Supplementary file 1 — Supplementary Text Legends for movies S1 to S5 Legend for data file S1 [file sciadv.abj5689_sm.pdf]

Science Advances
AAAS

Supplementary Materials for

Crab in amber reveals an early colonization of nonmarine environments during the Cretaceous

Javier Luque*, Lida Xing*, Derek E. G. Briggs, Elizabeth G. Clark, Alex Duque, Junbo Hui, Huijuan Mai, Ryan C. McKellar

*Corresponding author. Email: jluque@fas.harvard.edu, luque@ualberta.ca (J.L.); xinglida@gmail.com (L.X.)

Published 20 October 2021, *Sci. Adv.* **7**, eabj5689 (2021)
DOI: 10.1126/sciadv.abj5689

The PDF file includes:

Supplementary Text
Legends for movies S1 to S5
Legend for data file S1

Other Supplementary Material for this manuscript includes the following:

Movies S1 to S5
Data file S1

Supplementary Text

Systematic remarks

Our phylogenetic analysis recovered *Cretapsara athanata* Luque gen et sp. nov. nested within modern-looking eubrachyurans. In the Bayesian inference (BI) consensus tree, Eubrachyura was recovered as a well-supported clade with a high posterior probability (Fig. 5). Within Eubrachyura, Dorippidae (Fig. 5A) is recovered as the least inclusive of the crown group studied, with dorippids and extinct forms such as Telamonocarcinidae (Fig. 5B) and Archaeochiapasidae (Fig. 5C) recovered as sequential sister taxa to a well-supported clade formed by the remainder of the eubrachyuran ingroup, including *C. athanata* (Fig. 5D–T).

In the BI consensus tree, *Cretapsara* (Fig. 5F) is recovered in a trichotomy together with a poorly supported Componocancridae (Fig. 5D) + Marocarcinidae (Fig. 5E) clade, and the remainder of the eubrachyuran ingroup. The 'higher' eubrachyurans such as the freshwater groups Trichodactylidae (Fig. 5G) and Pseudothelphusidae (Fig. 5M), and a well-supported clade formed by the families Potamonautidae, Potamidae, and Gecarcinucidae (Fig. 5N–P), are collapsed into a soft polytomy together with the clades formed by the portunoids and relatives (Fig. 5H–K), and Thoracotremata (Fig. 5Q–T). In the BI topology, the heterotreme portunoid families †Eogeryonidae (Fig. 5H), Carcinidae (Fig. 5I), Geryonidae (Fig. 5J), and Portunidae (Fig. 5K), form a clade with moderate support, as does the Thoracotremata clade represented in our dataset by Grapsidae, Sesarmidae, Gecarcinidae, and Ocypodidae (Fig. 5Q–T).

In our analysis, and similar to most other molecular and morphological phylogenetic works (see main text for references), the podotreme brachyurans are recovered as a paraphyletic grouping, with Raninoidea (Lyreididae + Raninidae), Cyclodorippoidea (Cyclodorippidae + Cynomnidae), and Eubrachyura recovered forming a clade with one of the highest support values (Fig. 5).

Movie S1.

Rotational video of *Cretapsara athanata* Luque gen. et sp. nov., holotype LYAM-9, 3D mesh reconstruction. Video by Elizabeth G. Clark.

Movie S2.

Rotational video of micro-CT scanning of *Cretapsara athanata* Luque gen. et sp. nov., holotype LYAM-9, translucent rendering highlighting gills. Video by Elizabeth G. Clark.

Movie S3.

Rotational video of micro-CT scanning of *Cretapsara athanata* Luque gen. et sp. nov., holotype LYAM-9, translucent rendering focused on details of gills. Video by Elizabeth G. Clark.

Movie S4.

Rotational video of micro-CT scanning of *Cretapsara athanata* Luque gen. et sp. nov., holotype LYAM-9 gill structure in isolation. Video by Elizabeth G. Clark.

Movie S5.

Artistic video animation of *Cretapsara athanata* Luque gen. et sp. nov. holotype LYAM-9 extracted from surrounding amber and placed in inferred freshwater or brackish shoreline habitat near amber-producing forest. Video by Alex Duque.

Data file S1.

3D polygonal mesh of reconstructed 3D model of *Cretapsara athanata* Luque gen. et sp. nov., based on holotype LYAM-9, and used in the Supplemental Movie S5. Data file by Alex Duque.
